# An Optimization-Driven Analysis Pipeline to Uncover Biomarkers and Signaling Paths: Cervix Cancer

**DOI:** 10.3390/microarrays4020287

**Published:** 2015-05-28

**Authors:** Enery Lorenzo, Katia Camacho-Caceres, Alexander J. Ropelewski, Juan Rosas, Michael Ortiz-Mojer, Lynn Perez-Marty, Juan Irizarry, Valerie Gonzalez, Jesús A. Rodríguez, Mauricio Cabrera-Rios, Clara Isaza

**Affiliations:** 1 Bio IE Lab, The Applied Optimization Group at UPRM, Industrial Engineering Department, University of Puerto Rico at Mayaguez, Call Box 9000, Mayagüez, PR 00681, USA; E-Mails: enery.lorenzo@upr.edu (E.L.); katia.camacho@upr.edu (K.C.-C.); juan.rosas1@upr.edu (J.R.); michael.ortiz6@upr.edu (M.O.-M.); juan.irizarry4@upr.edu (J.I.); valerie.gonzalez9@upr.edu (V.G.); jesusandres.rodriguez@upr.edu (J.A.R.); mauricio.cabrera1@upr.edu (M.C.-R.); 2 Pittsburgh Supercomputing Center, 300 S. Craig Street, Pittsburgh, PA 15213, USA; E-Mail: ropelews@psc.edu; 3 Department of Pharmacology and Toxicology, Ponce School of Medicine, PO Box 700, Ponce, PR 00732, USA

**Keywords:** traveling salesman problem, signaling pathways, cancer biology

## Abstract

Establishing how a series of potentially important genes might relate to each other is relevant to understand the origin and evolution of illnesses, such as cancer. High-throughput biological experiments have played a critical role in providing information in this regard. A special challenge, however, is that of trying to conciliate information from separate microarray experiments to build a potential genetic signaling path. This work proposes a two-step analysis pipeline, based on optimization, to approach meta-analysis aiming to build a proxy for a genetic signaling path.

## 1. Introduction

Technology advancement has accelerated the capability to generate large amounts of biological data. The capability to translate these data into usable knowledge has, however, grown at a much slower rate. The technologies used to generate these data are often rendered obsolete by newer ones before the data already available are fully analyzed and taken to their full potential for biological and medical advancement. Microarrays constitute a technology of this sort: one used to generate a large number of experiments, many of which will be greatly under-utilized. The analysis of microarrays, however, still holds a large potential for the discovery of genetic biomarkers for all types of cancer, as well as elicit their signaling pathways. Extracting this kind of knowledge from microarray experiments has historically been considered challenging, largely due to two main difficulties: (i) the use of incommensurable units across different experiments, and (ii) the lack of analysis techniques that converge to a consistent set of biomarkers. These two difficulties propagate uncertainty to the task of determining a reliable signaling pathway. To this end, this work proposes a two-step pipeline that involves (1) a meta-analysis strategy, based on multiple-criteria optimization, which circumvents both of the main difficulties described previously to detect highly differentially expressed genes; and (2) a method, based on integer programming to find the most correlated path among the genes from the previous step. The central hypothesis is that there is a strong signal of relative expression in microarrays that is effectively discoverable through mathematical optimization.

It is critical that the detection of genetic cancer biomarkers through meta-analysis can be carried out faster, more consistently, and more accurately in order to shorten the lead-time from data generation to data interpretation and knowledge application. The simultaneous meta-analysis of multiple experiments via optimization and the subsequent identification of the highest correlated genetic path described in this work offer these capabilities. Microarray data already in repositories can be readily analyzed and, prospectively, new high-throughput biological technologies could be fully utilized earlier in the fight against cancer. The gap between raw data and applicable biomedical/medical knowledge can be reduced significantly; especially when considering that historic biological data will now be able to be brought into perspective to design new experiments and focus on more precise aspects of exploration.

## 2. Method

The proposed analysis pipeline has two sequential stages: (1) Meta-analysis for detection of highly differentially expressed genes and (2) finding the most correlated path. These are explained next.

### 2.1. Stage 1: Meta-Analysis for Detection of Highly Differentially Expressed Genes

Meta-analysis involves the joint study of multiple databases to obtain conclusions that apply across all of them. Meta-analysis can help detect potential genetic cancer biomarkers through the study of microarray databases. However, to this end, a series of difficulties are apparent: (a) Microarray experiments that are publicly available use different technologies, platforms and, most of the times, different scales. Incommensurability renders many meta-analyses efforts unfeasible [1] due to the inability to make comparisons across all experiments of interest. Even when the same units are used, often time, data normalization is required for comparability. (b) There is not an efficient, systematic method to carry out meta-analysis. Most of the studies analyze one particular database and try to generalize the results to other databases or analyze several databases separately and try to make sense of all the independent results [2]. (c) The issue of having a large number of measurements and genes generally results in large number of significant genes that must be validated [3]. (d) Meta-analysis of microarrays—and of high throughput biological experiments in general—is a laborious process that is often outpaced by the development of technology to generate ever-larger data sets. That is, data generation capabilities are larger and grow faster than our abilities to make sense and translate these data into usable knowledge. (e) Large repositories of public data generated through costly microarray experiments could go underanalyzed and underutilized in the fight for cancer when the researchers’ attention shifts to the next high-throughput technology. The problem of making sense of large quantities of data, however, will persist.

### 2.2. Multiple Criteria Optimization

**Figure 1:**
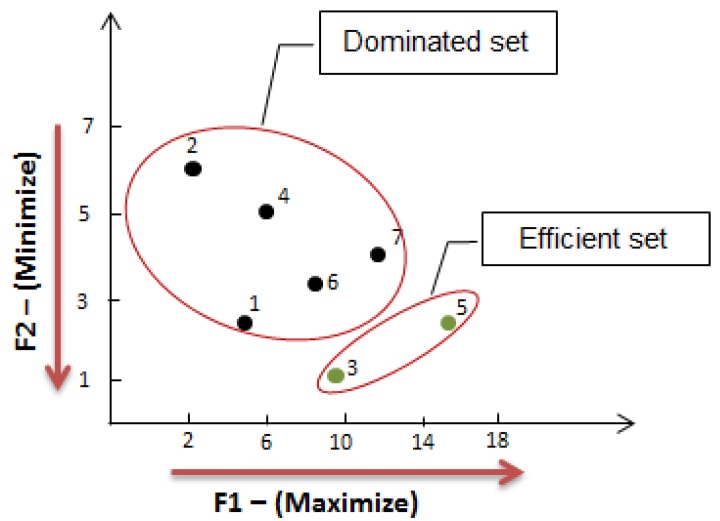
Representation of a multiple criteria optimization problem with two performance measures.

Multiple Criteria Optimization (MCO) is a field from Engineering Mathematics that deals with making decisions in the presence of multiple performance measures in conflict, *i.e.*, decisions where optimizing one criterion results in moving away from optimality in at least another criterion. Because of the presence of conflict, an MCO problem does not find a single best solution but rather a set of best compromising solutions in light of the performance measures under analysis. The best compromises define solutions called Pareto-Efficient (or simply Efficient, for short) that define the Efficient Frontier of the MCO problem at hand. A typical multiple criteria optimization with two conflicting performance measures (objectives), PMs, can be visualized as in Figure 1. In this figure, a set of seven candidate points, characterized by their values on both performance measures, are shown. The performance measure represented in the x-axis is to be maximized while the performance measure in the y-axis is to be minimized in this example. The problem is to find those candidate points that dominate all of the other points in both performance measures. In the face of conflict, this will result in a group of candidates in the southeast extreme of the set in Figure 1, solutions 3 and 5. These are Pareto-efficient solutions and, when all of them are accounted for, they integrate the Efficient Frontier of the MCO problem. In this example, it can be noted that among efficient solutions, an improvement in one performance measure can only come strictly at the detriment of another one: moving from solution 5 to solution 3 will result in an improvement in the performance measure associated to the vertical direction, but in a loss in the performance measure associated to the horizontal direction. Note that the general problem involves at least two performance measures to be optimized, where only the case with two performance measures has a convenient graphical representation. An MCO problem, however, can include as many dimensions (or performance measures) as necessary.

The general mathematical formulation of an unconstrained MCO problem is as follows:

(1) $$\begin{array}{l} Find\;x\;to\\ Minimize\;f_{j}\left(x\right)\;\;\;\;\;j=1,2,\ldots ,J \end{array}$$

The MCO problem in (1) can be discretized onto a set K  with |K| points in the space of the decision variables so as to define particular solutions $x^{k},\;\;(k=1,2,\ldots ,\;\left|K\right|)$ which can, in turn, be evaluated in the J performance measures to result in values $f_{j}\left(x^{k}\right)$. That is, the $k^{th}$ combination of values for the decision variables evaluated in the $j^{th}$ objective function. The illustrative example in Figure 1 follows this discretization with J=2 performance measures and |K|=7 solutions.

The MCO formulation under such discretization is, then as follows:

(2) $$\begin{array}{l} Find\;x^{k}\;\left(k\in K\right)\;to\\ Minimize\;f_{j}\left(x^{k}\right)\;\;\;\;\;j=1,2,\ldots ,J \end{array}$$

The solutions to (2) are, then, the Pareto-efficient solutions of the discretized MCO problem. Considering formulation (2), a particular combination $x^{0}$ with evaluations $f_{j}\left(x^{0}\right)$ will yield a Pareto-Efficient solution to (2) if and only if no other solution $x^{\psi }$ exists that meets two conditions, from this point on called Pareto-optimality conditions:

(Condition 1) $$f_{j}\left(x^{\psi }\right)\leq f_{j}\left(x^{0}\right)\;\forall j$$

(Condition 2) $$f_{j}\left(x^{\psi }\right)<f_{j}\left(x^{0}\right)\;in\;at\;least\;one\;j$$

Conditions (1) and (2) imply that no other solution $x^{\psi }$ dominates the solution under evaluation, $x^{0}$, in all performance measures simultaneously.

In previous publications [3,4] our group has demonstrated that if a set of candidate solutions evaluated by multiple performance measures is available, it is possible to determine a series of best compromises between all criteria through a technique called Data Envelopment Analysis (DEA). The idea behind DEA is to use an optimization model to compute a relative efficiency score for each particular solution with respect to the rest of the candidate solutions. The resulting best compromises, identified through their efficiency score, form the envelope of the solution set, therefore the name Data Envelopment Analysis. These solutions are indeed Pareto-efficient solutions of the problem under analysis.

The DEA linear programming formulations proposed by Banks, Charnes, and Cooper [5] are shown below:

(3) $$\begin{array}{l} Find\;\mu ,\;\nu ,\;\mu_{0}^{+},\mu_{0}^{-}\;to\\ Maximize\;\mu^{T}Y_{0}^{max}+\;\;\mu_{0}^{+}-\mu_{0}^{-}\\ Subject\;to\\ \;\nu^{T}Y_{0}^{min}=1\\ \mu^{T}Y_{j}^{max}-\;\nu^{T}Y_{j}^{min}+\mu_{0}^{+}-\mu_{0}^{-}\leq 0\;\;\;j=1,\ldots ,n\\ \;\mu^{T}\geq \varepsilon \cdot 1\\ \;\nu^{T}\geq \varepsilon \cdot 1\\ \;\mu_{0}^{+},\mu_{0}^{-}\;\geq 0 \end{array}$$

(4) $$\begin{array}{l} Find\;\mu ,\;\nu ,\nu_{0}^{+}-\nu_{0}^{-}\;\;\;\;\;to\\ Minimize\;\nu^{T}Y_{0}^{max}+\;\;\nu_{0}^{+}-\nu_{0}^{-}\\ Subject\;to\\ \;\mu^{T}Y_{0}^{max}=1\\ \;\;\nu^{T}Y_{j}^{min}-\mu^{T}Y_{j}^{max}+\nu_{0}^{+}-\nu_{0}^{-}\geq 0\;\;\;j=1,\ldots ,n\\ \;\nu^{T}\geq \varepsilon \cdot 1\\ \;\mu^{T}\geq \varepsilon \cdot 1\\ \;\nu_{0}^{+},\nu_{0}^{-}\;\geq 0 \end{array}$$

where **μ** and **ν** are column vectors containing multipliers to be optimally determined together with scalar variables $\mu_{0}^{+}$ and $\mu_{0}^{-}$ in the first case and together with $\nu_{0}^{+}$ and $\nu_{0}^{-}$ in the second case; $Y_{j}^{\min}$ and $Y_{j}^{\max}$ are column vectors containing the values of the *j*th combination of performance measures to be minimized and maximized respectively; and *ε* is a scalar usually set to a value of 1 × 10^−6^.

**Figure 2:**
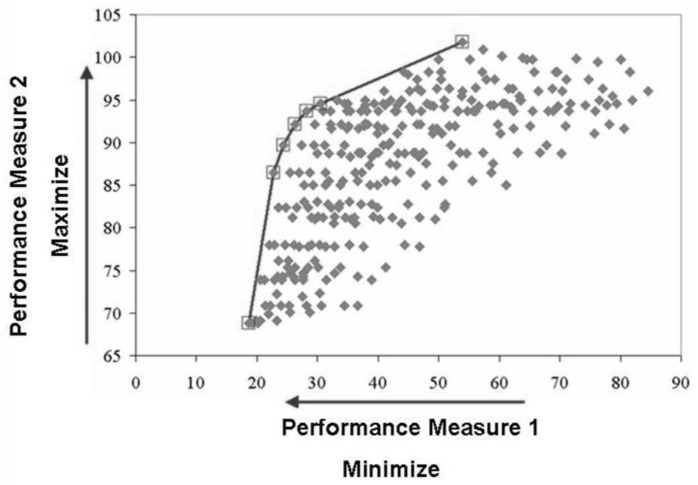
Multiple Criteria Optimization Problem solved using Data Envelopment Analysis (BCC model). The efficient solutions are identified through the use of piecewise-linear segments.

Model (3) is called the BCC Input Oriented Model and Model (4) is called the BCC Output Oriented Model. Both models are applied to each of the *n* candidate solutions. A particular solution with an objective function score of 1 (*i.e.*, an efficiency score of 1) using both formulations is in the envelope of the set and is considered to be an efficient solution to the MCO problem. The BCC model is just one of many possible DEA formulations, albeit a very powerful one. This model’s mathematical linear nature provides it with the capability of finding efficient solutions associated with the data set under analysis through a series of piece-wise linear segments. Nonlinear behavior is, then, approached with tractability and with the certainty that at least the efficient solutions lying in the convex part of the frontier are being found. Figure 2 shows an MCO problem solved through with DEA, specifically with the BCC model.

**Figure 3:**
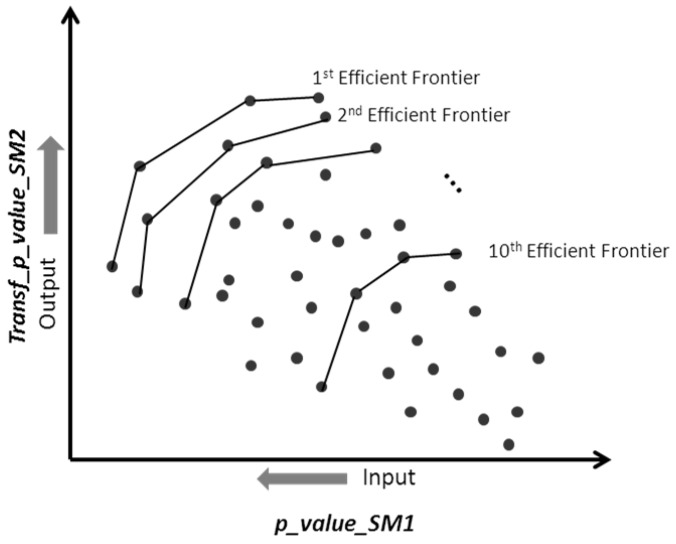
A case with genes characterized by two performance measures. Referring to this figure, and following the proposed method, at this point it is recommended to identify the first 10 efficient frontiers. This can be easily done by identifying the genes in the first efficient frontier through DEA, then removing them from the set and continue with a second DEA iteration.

DEA has several advantages including: (i) computational efficiency owing to its linear optimization structure; (ii) objectivity and consistency of results, which follows from not requiring the adjustment of parameters or assigning weights to the different performance measures by the user, and (iii) capability of analyzing several microarray experiments with incommensurate units. Appendix A discusses the volcano plot, a widely used tool to detect differentially expressed genes, to illustrate how the analyst can bias the results. On the other hand, one limitation of DEA is that of depending on a series of local linear approximations, as shown in Figure 2. Every time that a linear segment is superimposed over the set under analysis, there are genes lying in the nonconvex part of the set frontier that escape detection. These genes could be potential biomarkers, however. In order to circumvent the said disadvantage, the authors proposed that DEA be applied successively 10 times, each time removing the genes found in a particular iteration from the set for subsequent analyses. This strategy results in 10 frontiers, as seen in Figure 3. The number of efficient frontiers is, admittedly, an arbitrary number at this point, thus further refinement is necessary in this aspect.

At the end of Stage 1, the analyst is left with a set of differentially expressed genes that can be investigated to establish their role in the condition or illness under study, cancer in this case. This set of genes in the proposed method, however, will be used to determine how these are maximally correlated in Stage 2.

### 2.3. Stage 2: Finding the Most Correlated Path

It is proposed that the most correlated path among the list of candidate genes identified in the previous stage can be found optimally. To this end, the optimization problem identified in the literature as the Travelling Salesman Problem (TSP), is introduced here as a viable model.

The TSP is generally stated as follows: a salesman needs to visit n cities and needs to minimize the travel distance starting and finishing in the city of origin. Each city must be visited only once. The solution, then, is a tour. In n cities, there is a total of n! tours. If a particular city of origin is selected *a priori*, then the number of tours is (n-1)!. In our case, the objective is to find the tour among n genes of interest that maximizes the sum of the absolute values of pairwise correlations. This tour would then be interpreted as a surrogate for a biological pathway, defined as “a series of actions among molecules in a cell” [6], and more specifically for a genetic signaling pathway. A biological pathway “can provide clues about what goes wrong when a disease strikes.” [6].

**Figure 4:**
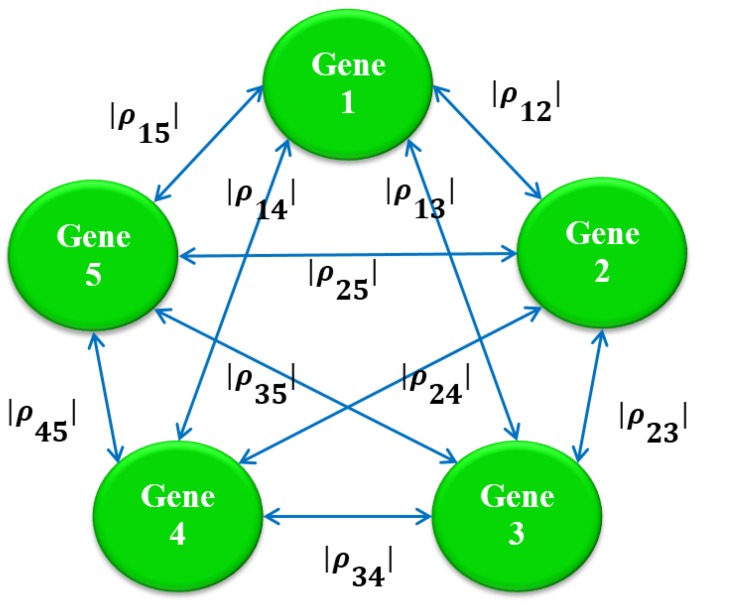
Representation of the many options for a cyclic path for 5 genes.

As a first approximation, it is proposed that the absolute values of linear correlation coefficients computed among a list of genes of potential biomarkers be used to construct networks such as the one presented in Figure 4, where the TSP can be readily applied. The idea of using a linear statistical correlation is, indeed, widely used in the literature as a means to uncover genetic coexpression. This information, in turn, should help cancer researchers in understanding the disease as well as look for targeted treatments. The paper by Kumari *et al.* [7] has studied different coexpression measurements, recommending to carry out a preliminary study to determine the most appropriate one for different objectives. It is, then, convenient at this point to resort to the use of the Pearson correlation coefficient as a starting point in this work.

The TSP can, indeed, be understood as an optimization problem. Consider that *c_ij_* represents the cost of traveling from city *i* to city *j* and let *y_ij_* be a binary variable, indicating whether or not the salesman travels from city *i* to city *j*. Additionally let us define flow variables *x_ij_* on each arc (*i,j*) and assume that the salesman has *n*-1 units available at node 1, which is arbitrarily selected as a “source node”, and he must deliver 1 unit to each of the other nodes [7]. The optimization model is as follows:

(5a) $$Minimize\;\underset{\left(i,j\right)\in A}{\sum }c_{ij}y_{ij}$$

(5b) $$\underset{1\leq j\leq n}{\sum }y_{ij}=1\;\;\;\;\;\;\forall i=1,2,\ldots ,n$$

(5c) $$\underset{1\leq i\leq n}{\sum }y_{ij}=1\;\;\;\;\;\forall \;j=1,2,\ldots ,n$$

(5d) Nx=b

(5e) $$x_{ij}\leq \left(n-1\right)y_{ij}\;\;\;\forall \left(i,j\right)\in A$$

(5f) $$x_{ij}\geq 0\;\;\;\;\;\;\;\forall \left(i,j\right)\in A$$

(5g) $$y_{ij}=0\;or\;1\;\;\;\;\;\;\;\forall \left(i,j\right)\in A$$

Following the description in [8], let *A’* = {(*i*,*j*): *y_ij_* =1} and let *A’’* ={(*i,j*): *x_ij_* >0}. The constraints (5b) and (5c) imply that exactly one arc of *A’* leaves and enters any node *i*; therefore, *A’* is the union of node disjoint cycles containing all of the nodes of N. In general, any integer solution satisfying (5b) and (5c) will be a union of disjoint cycles; if any such solution contains more than once cycle; they are referred to as subtours, since they pass through only a subset of nodes.

In constraint (5d) N is an *nxm* matrix, called the *node-arc incidence matrix* of the minimum cost flow problem. Each column N_ij_ in the matrix corresponds to the variable *x_ij_*. The column N_ij_ has a +1 in the *i*th row, a −1 in the *j*th row; the rest of its entries are zero. Constraint (5d) ensures that *A”* is connected since we need to send 1 unit of flow from node 1 to every other node via arcs in *A”*. The forcing constraints (5e) imply that *A”* is a subset *A’*. These conditions imply that the arc set *A’* is connected and thus cannot contain subtours [8].

The TSP is known to be a hard problem to solve to optimality; however, with a manageable number of entities (nodes) optimality is well within reach. In our group’s experience it has been possible to obtain the optimal TSP tour with a list of up to 100 genes in less than 1 hour of computing time in a personal computer. The Branch and Bound—an exact algorithm—was used to this end, as coded in Matlab. An exact algorithm is defined as one capable to arrive to a global optimal solution—provided that one exists—with certainty. Although it is also possible to use heuristics to approach the TSP, it must be understood that a heuristic method by definition does not provide certainty on arriving to a global optimal solution.

Referring back to Figure 4, it should be now apparent that in n genes associated to the nodes in the network, it is possible to obtain pairwise correlations to connect all genes among them resulting in a fully connected network. This network, in turn, can be mathematically translated into formulation (5a)–(5g) to identify the most correlated path. Thus, at the end of this stage, the most correlated path among all candidate genes from Stage 1, will be available as a proxy for a signalling path. The application of this two-stage analysis pipeline is demonstrated next in the context of cervix cancer.

## 3. Results for Cervix Cancer

### 3.1. Stage 1

In order to demonstrate the proposed analysis pipelines, this section presents results in cervix cancer previously published in [3]. The database used for this study was introduced in [9] and contained 8 healthy tissues and 25 cervical cancer tissues, all of them with expression level readings for 10,692 genes from a cDNA microarray. The list of 28 potential biomarkers after applying DEA is shown in Table 1. The genes in this list were cross validated for agreement in the direction of expression change in an independent database associated to [10]. As described previously, these genes were extracted from the first 10 frontiers of the analysis. The role of the selected genes in cancer was previously discussed in a previous publication of our group [3]. The fourth column of Table 1 summarizes the types of cancer that where the particular genes were found to be involved following such results.

**Table 1:** List 28 genes found through DEA as being differentially expressed in cervix cancer and cross validated for the direction of expression change [3].

Gene Probe	Gene Name	Sign of expression change from healthy tissues to cancer tissues		Examples of cancer types where the gene is involved	Reference
		Database 1 [8]	Database 2 [9]		
202575_at	CRABP2	-	-	Head and Neck, Breast	[11,12]
205402_x_at	PRSS2	-	-	Colorectal, Gastric Tumorigenesis	[13,14]
218677_at	S100A14	-	-	Esophageal squamous cell carcinoma cells, oral squamous cell carcinoma	[15,16]
202096_s_at	TSPO	-	-	Thyroid, Breast	[17,18]
212249_at	PIK3R1	-	-	Endometrial, Colorectal	[19,20]
212567_s_at	MAP4	-	-	Breast, non small cell lung carcinomas	[21,22]
211366_x_at	CASP1	-	-	Cervical squamous carcinoma cells	[23]
212889_x_at	GADD45GIP1	-	-	SKOV3 and HeLa cell lines	[24]
206626_x_at	SSX1	-	-	Prostate, multiple myeloma	[25,26]
213450_s_at	ICOSLG	-	-	Metastatic melanoma, ductal pancreatic adenocarcinoma	[27,28]
220405_at	SNTG1	-	-		
208032_s_at	GRIA3	-	-	Pancreatic	[29]
205690_s_at	BUD31	-	-		
206543_at	SMARCA2	-	-	Prostate, Skin	[30,31]
212291_at	HIPK1	+	+	Acute myeloid leukemia	[32,33]
211615_s_at	LRPPRC	+	+	Lung adenocarcinoma cell lines, oesophageal squamous cell carcinoma, stomach, colon, mammary and endometrial adenocarcinoma, and lymphoma	[34]
222027_at	NUCKS1	+	+	Breast	[35]
205362_s_at	PFDN4	+	+	Colorectal	[36]
211929_at	HNRNPA3	+	+	Non-small cell lung cancer	[37]
203738_at	C5orf22	+	+		
201794_s_at	SMG7	+	+		
200607_s_at	RAD21	+	+	Breast	[38]
201011_at	RPN1	+	+	Hematologic malignancies	[39]
201761_at	MTHFD2	+	+	Bladder, breast	[40,41]
203880_at	COX17	+	+	Non-small cell lung cancer	[42]
212255_s_at	ATP2C1	+	+	Breast, Cervical	[43,44]
205112_at	PLCE1	+	+	Gastric adenocarcinoma, colorectal	[45,46]
201663_s_at201664_at	SMC4	+	+	Breast, cervical	[9,47,48]

### 3.2. Stage 2

**Figure 5:**
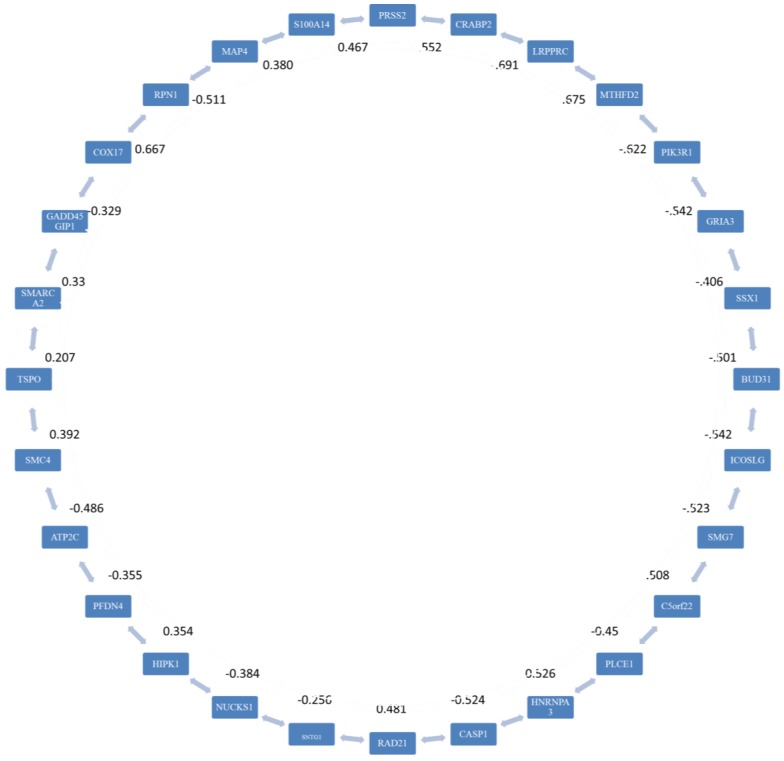
Highest Correlated Cyclic Path among the 28 genes identified in Stage 1.

Correlation is used in this project as a proxy for inhibitory or excitatory behavior between differences in the expression levels of two genes. As a first step, the linear correlation values between potential biomarkers are obtained. The following step was to arrange the correlation values in a matrix. To construct this matrix, first the differences between control and cancer tissues had to be calculated for each gene. Then, the absolute values of the correlation coefficients were calculated among each pair of genes based on these differences and stored in the said matrix. The absolute correlation values were consequently associated to the arcs in a fully connected graph with nodes representing potential biomarker genes. The resulting graph made possible the use of the formulation of the TSP. The optimal solution to this particular TSP is the tour among the genes of interest with the largest possible correlation, or similarly, the most correlated cyclic path as shown in Figure 5. It must be recalled at this point that there are a total of 28! $\approx 3.04\;\times 10^{29}\;$ ways in which a cyclic path can be drawn among the 28 genes.

The TSP formulation allows a wide range of analyses. In this case, the idea was to test the stability of the TSP solutions. In order to do so, TSP solutions were obtained using increasing numbers of potential biomarkers in the list of genes presented in Table 1 following the increasing order of the efficient frontier in which these were found. Starting with five genes, each time five more genes were introduced until the list was depleted on each case. Path segments that persisted across both databases were identified. Furthermore, path segments that persisted along the entire study were deemed the most stable. The results of this study were then matched against known biological pathways publicly available in the Kyoto Encyclopedia of Genes and Genomes (KEGG) [49]. A python script was written to make this process more efficient. This script is provided in Appendix B. Table 2 summarizes the results for each progressive analysis that introduced five genes at a time. As shown in Table 2, (LRPPRC with MTHFD2) and (RPN1 with COX17) are adjacent in the correlated cyclic path when the optimal solution is obtained for 25 and 28 genes. In addition, gene S100A14 is adjacent to TSPO when the optimal solution for 5, 15, 20, and 25 genes is found.

**Table 2:** Adjacent genes in the solutions for the correlated cyclic path found adding five genes at a time.

Number of Genes	Adjacent Genes
5	(CRABP2 with PRSS2) and (S100A14 with TSPO)
10	(PIK3R1 with MAP4) and (GADD45GIP1 with ICOSLG)
15	(SSX1 with BUD31), (ICOSLG with SNTG1), and (S100A14 with TSPO)
20	(LRPPRC with C5orf22) and (S100A14 with TSPO)
25	(S100A14 with TSPO), (SSX1 with GRIA3), (LRPPRC with MTHFD2), (RAD21 with BUD31), and (RPN1 with COX17)
28	(LRPPRC with MTHFD2) and (RPN1 with COX17)

A search for biological pathways in KEGG databases was conducted, however not every gene could be linked to a pathway. When comparing the known biological pathways with the obtained optimal solutions, for database GSE 7803 [9] and GSE 9750 [10], the only genes that appeared adjacent in the correlated cyclic path for both were COX17 with RPN1 and the only KEGG pathway common to both has the identifier 01100 that corresponds to the collection of Methabolic pathways. On the other hand, for database GSE 7803 [9], medium correlation was observed between genes HNRPA3 with BUD31, and both gene products are present in KEGG pathway 03040 that corresponds to the splisosome. For database GSE 9750 [10], PLCE1 is adjacent to PIK3R1, both gene products share the following KEGG pathways: 04012 that corresponds to the ErbB signaling pathway, 04015 Ras signaling pathway, 04015 Rap1 signaling pathway, 04066 HIF-1 signaling pathway, 04070 Phosphatidylinositol signaling system, 04370 VEGF signaling pathway, 04650 Natural killer cell mediated cytotoxicity, 04660 T cell receptor signaling pathway, 04664 Fc epsilon RI signaling pathway, 04666 Fc gamma R-mediated phagocytosis, 04670 Leukocyte transendothelial migration, 04722 Neurotrophin signaling pathway, 04750 Inflammatory mediator regulation of TRP channels, 04919 Thyroid hormone signaling pathway, 05169 Epstein-Barr virus infection, 05200 Pathways in cancer, 05200 Pathways in cancer, 05214 Glioma, 05223 Non-small cell lung cancer, and the 05231 KEGG pathway that corresponds to Choline metabolism in cancer.

**Table 3:** Selected genes localization.

Gene	Location
HIPK1	1p13.2
NUCKS1	1q32.1
SMG7	1q25.3
CRABP2	1q21.3
S100A14	1q21.1
HNRNPA3	2q31.2
LRPPRC	2p21
MTHFD2	2p13.1
SMC4	3q26.1
ATP2C	3q22.1
RPN1	3q21.3
MAP4	3p21.31
COX17	3q13.33
C5orf22	5p13.3
PIK3R1	5q13.1
BUD31	7q22.1
PRSS2	7q34
SNTG1	8q11.21
RAD21	8q24.11
SSX1	Xp11.23
GRIA3	Xq25
PFDN4	20q13.2
CASP1	11q22.3
PLCE1	10q23.33
ICOSLG	21q22.3
GADD45G	19p13.2
SMARCA2	9p22.3
TSPO	22q13.31

In cancer there are chromosomal physical changes that produce gains or losses of certain genes. To explore if the position of the genes in the cyclic path could also provide information about these chromosomal changes, the location of each gene was consider (this information was obtained from [50]). This information is listed in Table 3. All chromosomes in Table 3 have been reported as having changes in cervical cancer, in regions close to the ones where the selected genes belong. It is interesting to note that some of the genes that are neighbors in the cyclic path are also neighbors in their genetic localization.

HIPK1, NUCKS1, SMG7, and CRABP2 are all in chromosome 1, the first two genes of the list are adjacent in the cyclic path and the others are scatter through the cycle. Reported changes in chromosome 1 in cervical cancer include: gains in the 1p region [51,52,53], increment on the 1q32.1–32.2 genes expression [44], aneusomy of the chromosome [54] among others.

Three genes are in chromosome 2, HNRNPA3, LRPPRC and MTHFD2. There are several changes in chromosome 2 related to cervical cancer, for example reduced expression of genes in 2p has been reported [55], it has also been reported that deletions of the 2q33–q37 are common in cervical carcinoma [56] as well as loss of heterozygosity at 2q35–q37.1 [57].

COX17, RNP1, MAP4, and SMC4 (separated by three genes from the group), and ATP2C (adjacent to SMC4) are all in chromosome 3. Changes in chromosome 3 have been extensively reported for cervical cancer. Gain of chromosome 3q has been reported in pre-cancer and cancer of the cervix (these are some of the reports: [58,59,60,61]) while loss of 3p12-p14 has also been observed [62] and loss of heterozygosity on chromosome 3p has been also reported in this cancer [55].

C5orf22 and PIK3R1 are both in chromosome 5. Chromosome 5 is known to have alterations in cervical cancer [61,63,64,65]. BUD31 and PRSS2 belong to chromosomes 7, there are known changes of this chromosome in cervical cancer [66,67,68]. SNTG1 and RAD21 are in chromosome 8, examples of reported changes in this chromosome can be found in: [69,70,71,72]. Genes SSX1 and GRIA3 are both in X chromosome. Examples of the association of changes in chromosome X in cervical cancer can be found in [73,74,75]. Genes PFDN4, CASP1, PLCE1, ICOSLG, GADD45G, SMARCA2, and TSPO are located in different chromosomes, and there are reports for changes in each one of these chromosomes in cervix cancer, for examples the reader is refer to: [10,61,76,77,78,79,80,81,82,83,84,85,86,87,88,89].

The results suggest that the chromosomal gains and losses known for cervical cancer could include bigger regions. It is clear that true experimental validation is critical to further support the results of the proposed pipeline analysis at this point. It is also important, however, to notice its potential for biological discovery. Every time that a biological pathway is discovered, it basically is a problem of selecting a path by systematically choosing pairs of genes with scientific basis. If a mathematical point of view is adopted, this practice implies that the solution is built heuristically as opposed to optimally. This insight has important implications for the adoption of optimization methods in Medicine and Biology.

## 4. Conclusions

This work proposes a pipeline analysis based on optimization to facilitate the discovery of genetic signaling paths related to cancer and also could provide information about expanded chromosomal regions that are compromised for cases to be studied. In this instance, the method was applied to cervix cancer. The potential of the proposed method is significant if the detection of a biological pathway is understood as a combinatorial problem similar to the Traveling Salesman Problem, for which an optimal solution exists. If positively verified, this point of view could also imply that current biological pathways might have room for improvement to fully capture the signal in microarray experiments, and thus open the possibility of further discovery in the understanding—and fight—against cancer.

## Figures and Tables

**Figure A1:**
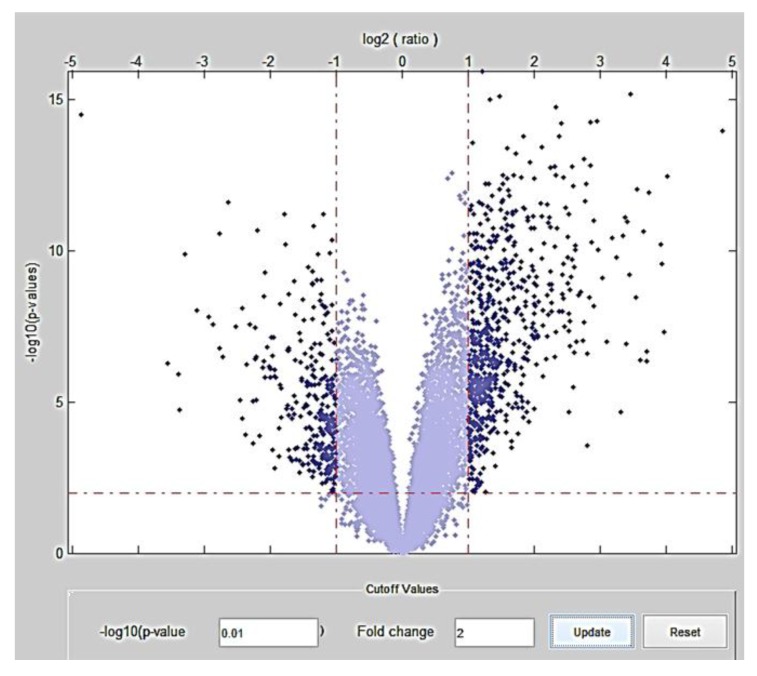
A volcano plot. Two cutoff values must be set by the user to decide upon the significance of different genes: one for fold change (x-axis) and one for *p*-value (y-axis).

## Appendix A

Stage 1 of the proposed analysis pipeline involves the use of Multiple Criteria Optimization (MCO). MCO does not require the adjustment of parameters by the users to detect differentially expressed genes, thus it preserves the objectivity and the consistent convergence of the analysis. As a comparative example, a tool like the volcano plot (Figure A1), would require for the user to define different cutoff values to select different genes, thereby biasing the analysis.

In addition to the evident result of choosing different sets of genes when the analyst picks different sets of cutoff values, this decision also greatly affects the number of genes that are deemed to change their expression significantly, as shown in Table A1.

**Table A1:** An example of how the number of genes deemed significant changes when choosing different cutoff values for fold change and *p*-value.

*p*-value	Fold change	Differentially expressed genes (number)	Number of genes Overexpressed	Number of genes Underexpressed
10^−2^	2	934	645	289
10^−2^	8	29	23	6
10^−2^	24	2	1	1
10^−7^	2	649	516	133
10^−7^	8	27	22	5
10^−7^	24	2	1	1
10^−12^	2	130	121	9
10^−12^	8	12	11	1
10^−12^	24	2	1	1

## Appendix B

A Python script was written to gather information from KEGG. This is provided in its three parts below.

**Part 1** #!/opt/python2.6/bin/python # Import Comma Separated Value Library ... import csv import sys import urllib2 # Open file to be read ifile = open('results.csv', "rb") # Create the reader object (in order to read from CSV file) reader = csv.reader(ifile) # Create file to be output ofile = open('details.csv', 'w') # Create writer in order to write to output file writer = csv.writer(ofile, delimiter=',', quotechar='"', quoting=csv.QUOTE_MINIMAL) # Function in charge of extracting each genes in the database file def detailPathwayExtractor(): for row in reader: if (len(row)==1): path=row[1] try: url_to_go_to = "http://rest.kegg.jp/get/" + path print url_to_go_to handle = urllib2.urlopen(url_to_go_to) #read contenent content = handle.read() for row_of_file in content.split("\n"): if row_of_file.split() != []: print row_of_file.split() writer.writerow(row_of_file.split()) # Run the pathway extractor function except IOError: print "can't open file" detailPathwayExtractor() # Close both opened files ifile.close() ofile.close()

**Part 2** #!/opt/python2.6/bin/python # Import Comma Separated Value Library ... import csv import sys import urllib2 # Open file to be read ifile = open('test.csv', "rb") # Create the reader object (in order to read from CSV file) reader = csv.reader(ifile) # Create file to be output ofile = open('results.csv', 'w') # Create writer in order to write to output file writer = csv.writer(ofile, delimiter=',', quotechar='"', quoting=csv.QUOTE_MINIMAL) # Function in charge of extracting each genes in the database file def pathwayExtractor(): for row in reader: if (len(row)==4): HSA=row[3] try: url_to_go_to = "http://rest.kegg.jp/link/pathway/hsa:" + HSA print url_to_go_to handle = urllib2.urlopen(url_to_go_to) #read contenent content = handle.read() for row_of_file in content.split("\n"): if row_of_file.split() != []: print row_of_file.split() writer.writerow(row_of_file.split()) # Run the pathway extractor function except IOError: print "can't open file" pathwayExtractor() # Close both opened files ifile.close() ofile.close()

**Part 3** #!/opt/python2.6/bin/python # Import Comma Separated Value Library import csv import sys import urllib2 # Open file to be read ifile = open('results.csv', "rb") # Create the reader object (in order to read from CSV file) reader = csv.reader(ifile) # Create file to be output ofile = open('details.csv', 'w') # Create writer in order to write to output file writer = csv.writer(ofile, delimiter=',', quotechar='"', quoting=csv.QUOTE_MINIMAL) # Function in charge of extracting each genes in the database file def detailPathwayExtractor(): for row in reader: if (len(row)==1): path=row[1] try: url_to_go_to = "http://rest.kegg.jp/get/" + path print url_to_go_to handle = urllib2.urlopen(url_to_go_to) #read contenent content = handle.read() for row_of_file in content.split("\n"): if row_of_file.split() != []: print row_of_file.split() writer.writerow(row_of_file.split()) # Run the pathway extractor function except IOError: print "can't open file" detailPathwayExtractor() # Close both opened files ifile.close() ofile.close()

## References

[1] Fierro A.C., Vandenbussche F., Engelen K., Van de Peer Y., Marchal K. (2008). Meta analysis of gene expression data within and across species. Curr. Genomics.

[2] Owzar K., Barry W.T., Jung S.H. (2011). Statistical considerations for analysis of microarray experiments. Clin. Transl. Sci..

[3] Sánchez-Peña M.L., Isaza C.E., Pérez-Morales J., Rodríguez-Padilla C., Castro J.M., Cabrera-Ríos M. (2013). Identification of potential biomarkers from microarray experiments using multiple criteria optimization. Cancer Medicine.

[4] Watts-Oquendo E., Sánchez-Peña M., Isaza C.E., Cabrera-Ríos M. (2012). Potential colon cancer biomarker search using more than two performance measures in a multiple criteria optimization approach. P. R. Health Sci. J..

[5] Charnes A., Cooper W.W., Lewin A.Y., Seiford L.M. (1993). Data Envelopment Analysis: Theory, Methodology and Applications.

[6] National Human Genome Research Institute National Institute of Health. http://www.genome.gov/27530687.

[7] Kumari S., Nie J., Chen H.S., Ma H., Stewart R., Li X., Lu M.Z., Taylor W.M., Wei H. (2012). Evaluation of gene association methods for coexpression network construction and biological knowledge discovery. PLoS ONE.

[8] Ahuja R.K., Magnanti T.L., Orlin J.B. (1993). Network Flows: Theory, Algorithms, and Applications.

[9] Zhai Y., Kuick R., Nan B., Ota I., Weiss S.J., Trimble C.L., Fearon E.R., Cho K.R. (2007). Gene Expression Analysis of Preinvasive and Invasive Cervical Squamous Cell Carcinomas Identifies HOXC10 as a Key Mediator of Invasion. Cancer Res..

[10] Scotto L., Narayan G., Nandula S.V., Arias-Pulido H., Subramaniyam S., Schneider A., Kaufmann A.M., Wright J.D., Pothuri B., Mansukhani M. (2008). Identification of copy number gain and overexpressed genes on chromosome arm 20q by an integrative genomic approach in cervical cancer: Potential role in progression. Gene Chromosome. Canc..

[11] Calmon M.F., Rodrigues R.V., Kaneto C.M., Moura R.P., Silva S.D., Mota L.D., Pinheiro D.G., Torres C., de Carvalho A.F., Cury P.M. (2009). Epigenetic silencing of CRABP2 and MX1 in head and neck tumors. Neoplasia.

[12] Geiger T., Madden S.F., Gallagher W.M., Cox J., Mann M. (2012). Proteomic portrait of human breast cancer progression identifies novel prognostic markers. Cancer Res..

[13] Williams S.J., Gotley D.C., Antalis T.M. (2001). Human trypsinogen in colorectal cancer. Int. J. Cancer.

[14] Rajkumar T., Vijayalakshmi N., Gopal G., Sabitha K., Shirley S., Raja U.M., Ramakrishnan S.A. (2010). Identification and validation of genes involved in gastric tumorigenesis. Cancer Cell Int..

[15] Chen H., Yuan Y., Zhang C., Luo A., Ding F., Ma J., Yang S., Tian Y., Tong T., Zhan Q., Liu Z. (2012). Involvement of S100A14 Protein in Cell Invasion by Affecting Expression and Function of Matrix Metalloproteinase (MMP)-2 via p53-dependent Transcriptional Regulation. J. Biol. Chem..

[16] Sapkota D., Bruland O., Costea D.E., Haugen H., Vasstrand E.N., Ibrahim S.O. (2011). S100A14 regulates the invasive potential of oral squamous cell carcinoma derived cell-lines *in vitro* by modulating expression of matrix metalloproteinases, MMP1 and MMP9. Eur. J. Cancer.

[17] Klubo-Gwiezdzinska J., Jensen K., Bauer A., Patel A., Costello J., Burman K., Wartofsky L., Hardwick M.J., Vasko V.V. (2012). The expression of translocator protein in human thyroid cancer and its role in the response of thyroid cancer cells to oxidative stress. J. Endocrinol..

[18] Mukherjee S., Das S.K. (2012). Translocator protein (TSPO) in breast cancer. Curr. Mol. Med..

[19] Cheung L.W., Hennessy B.T., Li J., Yu S., Myers A.P., Djordjevic B., Lu Y., Stemke-Hale K., Dyer M.D., Zhang F. (2011). High Frequency of PIK3R1 and PIK3R2 Mutations in Endometrial Cancer Elucidates a Novel Mechanism for Regulation of PTEN Protein Stability. Cancer Discov..

[20] Nowakowska-Zajdel E., Mazurek U., Ziółko E., Niedworok E., Fatyga E., Kokot T., Muc-Wierzgoń M. (2011). Analysis of expression profile of gene encoding proteins of signal cascades activated by insulin-like growth factors in colorectal cancer. Int. J. Immunopathol. Pharmacol..

[21] Chen X., Wu J., Lu H., Huang O., Shen K. (2012). Measuring β-tubulin III, Bcl-2, and ERCC1 improves pathological complete remission predictive accuracy in breast cancer. Cancer Sci..

[22] Cucchiarelli V., Hiser L., Smith H., Frankfurter A., Spano A., Correia J.J., Lobert S. (2008). Beta-tubulin isotype classes II and V expression patterns in nonsmall cell lung carcinomas. Cell Motil. Cytoskeleton.

[23] Arany I., Ember I.A., Tyring S.K. (2003). All-trans-retinoic acid activates caspase-1 in a dose-dependent manner in cervical squamous carcinoma cells. Anticancer Res..

[24] Nakayama K., Nakayama N., Wang T.L., Shih I.M. (2007). NAC-1 controls cell growth and survival by repressing transcription of Gadd45GIP1, a candidate tumor suppressor. Cancer Res..

[25] Smith H.A., Cronk R.J., Lang J.M., McNeel D.G. (2011). Expression and immunotherapeutic targeting of the SSX family of cancer-testis antigens in prostate cancer. Cancer Res..

[26] Van Duin M., Broyl A., de Knegt Y., Goldschmidt H., Richardson P.G., Hop W.C., van der Holt B., Joseph-Pietras D., Mulligan G., Neuwirth R. (2011). Cancer testis antigens in newly diagnosed and relapse multiple myeloma: Prognostic markers and potential targets for immunotherapy. Haematologica.

[27] Fu T., He Q., Sharma P. (2011). The ICOS/ICOSL pathway is required for optimal antitumor responses mediated by anti-CTLA-4 therapy. Cancer Res..

[28] Tjomsland V., Spångeus A., Sandström P., Borch K., Messmer D., Larsson M. (2010). Semi mature blood dendritic cells exist in patients with ductal pancreatic adenocarcinoma owing to inflammatory factors released from the tumor. PLoS ONE.

[29] Ripka S., Riedel J., Neesse A., Griesmann H., Buchholz M., Ellenrieder V., Moeller F., Barth P., Gress T.M., Michl P. (2010). Glutamate receptor GRIA3—Target of CUX1 and mediator of tumor progression in pancreatic cancer. Neoplasia.

[30] Sun A., Tawfik O., Gayed B., Thrasher J.B., Hoestje S., Li C., Li B. (2007). Aberrant expression of SWI/SNF catalytic subunits BRG1/BRM is associated with tumor development and increased invasiveness in prostate cancers. Prostate.

[31] Moloney F.J., Lyons J.G., Bock V.L., Huang X.X., Bugeja M.J., Halliday G.M. (2009). Hotspot mutation of Brahma in non-melanoma skin cancer. J. Invest. Dermatol..

[32] Mougeot J.L., Bahrani-Mougeot F.K., Lockhart P.B., Brennan M.T. (2011). Microarray analyses of oral punch biopsies from acute myeloid leukemia (AML) patients treated with chemotherapy. Oral Surg. Oral Med. Oral Pathol. Oral Radiol. Endod..

[33] Aikawa Y., Nguyen L.A., Isono K., Takakura N., Tagata Y., Schmitz M.L., Koseki H., Kitabayashi I. (2006). Roles of HIPK1 and HIPK2 in AML1- and p300-dependent transcription, hematopoiesis and blood vessel formation. EMBO J..

[34] Tian T., Ikeda J.I., Wang Y., Mamat S., Luo W., Aozasa K., Morii E. (2012). Role of leucine-rich pentatricopeptide repeat motif-containing protein (LRPPRC) for anti-apoptosis and tumourigenesis in cancers. Eur. J. Cancer.

[35] Ziółkowski P., Gamian E., Osiecka B., Zougman A., Wiśniewski J.R. (2009). Immunohistochemical and proteomic evaluation of nuclear ubiquitous casein and cyclin-dependent kinases substrate in invasive ductal carcinoma of the breast. J. Biomed. Biotechnol..

[36] Miyoshi N., Ishii H., Mimori K., Nishida N., Tokuoka M., Akita H., Sekimoto M., Doki Y., Mori M. (2010). Abnormal expression of PFDN4 in colorectal cancer: A novel marker for prognosis. Ann. Surg. Oncol..

[37] Boukakis G., Patrinou-Georgoula M., Lekarakou M., Valavanis C., Guialis A. (2010). Deregulated expression of hnRNP A/B proteins in human non-small cell lung cancer: Parallel assessment of protein and mRNA levels in paired tumour/non-tumour tissues. BMC Cancer.

[38] Atienza J.M., Roth R.B., Rosette C., Smylie K.J., Kammerer S., Rehbock J., Ekblom J., Denissenko M.F. (2005). Suppression of RAD21 gene expression decreases cell growth and enhances cytotoxicity of etoposide and bleomycin in human breast cancer cells. Mol. Cancer Ther..

[39] Shimizu S., Suzukawa K., Kodera T., Nagasawa T., Abe T., Taniwaki M., Yagasaki F., Tanaka H., Fujisawa S., Johansson B. (2000). Identification of breakpoint cluster regions at 1p36.3 and 3q21 in hematologic malignancies with t(1;3)(p36;q21). Genes Chromosome. Canc..

[40] Andrew A.S., Gui J., Sanderson A.C., Mason R.A., Morlock E.V., Schned A.R., Kelsey K.T., Marsit C.J., Moore J.H., Karagas M.R. (2009). Bladder cancer SNP panel predicts susceptibility and survival. Hum. Genet..

[41] Xu X., Qiao M., Zhang Y., Jiang Y., Wei P., Yao J., Gu B., Wang Y., Lu J., Wang Z. (2010). Quantitative proteomics study of breast cancer cell lines isolated from a single patient: Discovery of TIMM17A as a marker for breast cancer. Proteomics.

[42] Suzuki C., Daigo Y., Kikuchi T., Katagiri T., Nakamura Y. (2003). Identification of COX17 as a therapeutic target for non-small cell lung cancer. Cancer Res..

[43] Grice D.M., Vetter I., Faddy H.M., Kenny P.A., Roberts-Thomson S.J., Monteith G.R. (2010). Golgi calcium pump secretory pathway calcium ATPase 1 (SPCA1) is a key regulator of insulin-like growth factor receptor (IGF1R) processing in the basal-like breast cancer cell line MDA-MB-231. J. Biol. Chem..

[44] Wilting S.M., de Wilde J., Meijer C.J., Berkhof J., Yi Y., van Wieringen W.N., Braakhuis B.J., Meijer G.A., Ylstra B., Snijders P.J. (2008). Integrated genomic and transcriptional profiling identifies chromosomal loci with altered gene expression in cervical cancer. Genes Chromosomes Cancer.

[45] Wang M., Zhang R., He J., Qiu L., Li J., Wang Y., Sun M., Yang Y., Wang J., Yang J. (2012). Potentially functional variants of PLCE1 identified by GWASs contribute to gastric adenocarcinoma susceptibility in an eastern Chinese population. PLoS ONE.

[46] Danielsen S.A., Cekaite L., Ågesen T.H., Sveen A., Nesbakken A., Thiis-Evensen E., Skotheim R.I., Lind G.E., Lothe R.A. (2011). Phospholipase C isozymes are deregulated in colorectal cancer--insights gained from gene set enrichment analysis of the transcriptome. PLoS ONE.

[47] Chang H., Jeung H.C., Jung J.J., Kim T.S., Rha S.Y., Chung H.C. (2011). Identification of genes associated with chemosensitivity to SAHA/taxane combination treatment in taxane-resistant breast cancer cells. Breast Cancer Res. Treat..

[48] Kulawiec M., Safina A., Desouki M.M., Still I., Matsui S., Bakin A., Singh K.K. (2008). Tumorigenic transformation of human breast epithelial cells induced by mitochondrial DNA depletion. Cancer Biol. Ther..

[49] KEGG: Kyoto Encyclopedia of Genes and Genomes. http://www.genome.jp/kegg/.

[50] Rebhan M., Chalifa-Caspi V., Prilusky J., Lancet D. (1998). GeneCards: A novel functional genomics compendium with automated data mining and query reformulation support. Bioinformatics.

[51] Wang J., Tai L.S., Tzang C.H., Fong W.F., Guan X.Y., Yang M. (2008). 1p31, 7q21 and 18q21 chromosomal aberrations and candidate genes in acquired vinblastine resistance of human cervical carcinoma KB cells. Oncol. Rep..

[52] Lee M., Nam E.S., Jung S.H., Kim S.Y., Lee S.J., Yoon J.H., Lee N.W., Jeon S., Choi J.S., Cho C.H. (2014). 1p36.22 region containing PGD gene is frequently gained in human cervical cancer. J. Obstet. Gynaecol. Res..

[53] Wilting S.M., Steenbergen R.D., Tijssen M., van Wieringen W.N., Helmerhorst T.J., van Kemenade F.J., Bleeker M.C., van de Wiel M.A., Carvalho B., Meijer G.A. (2009). Chromosomal signatures of a subset of high-grade premalignant cervical lesions closely resemble invasive carcinomas. Cancer Res..

[54] Cortés-Gutiérrez E.I.1., Dávila-Rodríguez M.I., Muraira-Rodríguez M., Said-Fernández S., Cerda-Flores R.M. (2005). Association between the stages of cervical cancer and chromosome 1 aneusomy. Cancer Genet. Cytogenet..

[55] Kozlowski L., Filipowski T., Rucinska M., Pepinski W., Janica J., Skawronska M., Poznanski J., Wojtukiewicz M.Z. (2006). Loss of heterozygosity on chromosomes 2p, 3p, 18q21.3 and 11p15.5 as a poor prognostic factor in stage II and III (FIGO) cervical cancer treated by radiotherapy. Neoplasma.

[56] Rao P.H., Arias-Pulido H., Lu X.Y., Harris C.P., Vargas H., Zhang F.F., Narayan G., Schneider A., Terry M.B., Murty V.V. (2004). Chromosomal amplifications, 3q gain and deletions of 2q33-q37 are the frequent genetic changes in cervical carcinoma. BMC Cancer.

[57] Edelmann J., Richter K., Hänel C., Hering S., Horn L.C. (2006). X chromosomal and autosomal loss of heterozygosity and microsatellite instability in human cervical carcinoma. Int. J. Gynecol. Cancer.

[58] Thomas L.K., Bermejo J.L., Vinokurova S., Jensen K., Bierkens M., Steenbergen R., Bergmann M., von Knebel Doeberitz M., Reuschenbach M. (2014). Chromosomal gains and losses in human papillomavirus-associated neoplasia of the lower genital tract—A systematic review and meta-analysis. Eur. J. Cancer.

[59] Wright T.C., Compagno J., Romano P., Grazioli V., Verma Y., Kershnar E., Tafas T., Kilpatrick M.W. (2015). Amplification of the 3q chromosomal region as a specific marker in cervical cancer. Am. J. Obstet. Gynecol..

[60] Policht F.A., Song M., Sitailo S., O'Hare A., Ashfaq R., Muller C.Y., Morrison L.E., King W., Sokolova I.A. (2010). Analysis of genetic copy number changes in cervical disease progression. BMC Cancer.

[61] Luhn P., Houldsworth J., Cahill L., Schiffman M., Castle P.E., Zuna R.E., Dunn S.T., Gold M.A., Walker J., Wentzensen N. (2013). Chromosomal gains measured in cytology samples from women with abnormal cervical cancer screening results. Gynecol. Oncol..

[62] Lando M., Wilting S.M., Snipstad K., Clancy T., Bierkens M., Aarnes E.K., Holden M., Stokke T., Sundfør K., Holm R. (2013). Identification of eight candidate target genes of the recurrent 3p12-p14 loss in cervical cancer by integrative genomic profiling. J. Pathol..

[63] Johnson L.G., Schwartz S.M., Malkki M., Du Q., Petersdorf E.W., Galloway D.A., Madeleine M.M. (2011). Risk of cervical cancer associated with allergies and polymorphisms in genes in the chromosome 5 cytokine cluster. Cancer Epidemiol. Biomarkers Prev..

[64] Scotto L., Narayan G., Nandula S.V., Subramaniyam S., Kaufmann A.M., Wright J.D., Pothuri B., Mansukhani M., Schneider A., Arias-Pulido H. (2008). Integrative genomics analysis of chromosome 5p gain in cervical cancer reveals target over-expressed genes, including Drosha. Mol. Cancer.

[65] Huang F.Y., Chiu P.M., Tam K.F., Kwok Y.K., Lau E.T., Tang M.H., Ng T.Y., Liu V.W., Cheung A.N., Ngan H.Y. (2006). Semi-quantitative fluorescent PCR analysis identifies PRKAA1 on chromosome 5 as a potential candidate cancer gene of cervical cancer. Gynecol. Oncol..

[66] Schrevel M., Gorter A., Kolkman-Uljee S.M., Trimbos J.B., Fleuren G.J., Jordanova E.S. (2011). Molecular mechanisms of epidermal growth factor receptor overexpression in patients with cervical cancer. Mod. Pathol..

[67] Thein A., Trková M., Fox M., Parrington J. (2000). The application of comparative genomic hybridization to previously karyotyped cervical cancer cell lines. Cancer Genet. Cytogenet..

[68] Mian C., Bancher D., Kohlberger P., Kainz C., Haitel A., Czerwenka K., Stani J., Breitenecker G., Wiener H. (1999). Fluorescence *in situ* hybridization in cervical smears: Detection of numerical aberrations of chromosomes 7, 3, and X and relationship to HPV infection. Gynecol. Oncol..

[69] Ferber M.J., Eilers P., Schuuring E., Fenton J.A., Fleuren G.J., Kenter G., Szuhai K., Smith D.I., Raap A.K., Brink A.A. (2004). Positioning of cervical carcinoma and Burkitt lymphoma translocation breakpoints with respect to the human papillomavirus integration cluster in FRA8C at 8q24.13. Cancer Genet. Cytogenet..

[70] Sokolova I., Algeciras-Schimnich A., Song M., Sitailo S., Policht F., Kipp B.R., Voss J.S., Halling K.C., Ruth A., King W. (2007). Chromosomal biomarkers for detection of human papillomavirus associated genomic instability in epithelial cells of cervical cytology specimens. J. Mol. Diagn..

[71] Bhattacharya N., Singh R.K., Mondal S., Roy A., Mondal R., Roychowdhury S., Panda C.K. (2004). Analysis of molecular alterations in chromosome 8 associated with the development of uterine cervical carcinoma of Indian patients. Gynecol. Oncol..

[72] Seng T.J., Low J.S., Li H., Cui Y., Goh H.K., Wong M.L., Srivastava G., Sidransky D., Califano J., Steenbergen R.D. (2007). The major 8p22 tumor suppressor DLC1 is frequently silenced by methylation in both endemic and sporadic nasopharyngeal, esophageal, and cervical carcinomas, and inhibits tumor cell colony formation. Oncogene.

[73] Dellas A., Torhorst J., Gaudenz R., Mihatsch M.J., Moch H. (2003). DNA copy number changes in cervical adenocarcinoma. Clin. Cancer Res..

[74] Marzano R., Corrado G., Merola R., Sbiroli C., Guadagni F., Vizza E., Del Nonno F., Carosi M., Galati M.M., Sperduti I., Cianciulli A.M. (2004). Analysis of chromosomes 3, 7, X and the EGFR gene in uterine cervical cancer progression. Eur. J. Cancer.

[75] Hopman A.H., Smedts F., Dignef W., Ummelen M., Sonke G., Mravunac M., Vooijs G.P., Speel E.J., Ramaekers F.C. (2004). Transition of high-grade cervical intraepithelial neoplasia to micro-invasive carcinoma is characterized by integration of HPV 16/18 and numerical chromosome abnormalities. J. Pathol..

[76] Tabach Y., Kogan-Sakin I., Buganim Y., Solomon H., Goldfinger N., Hovland R., Ke X.S., Oyan A.M., Kalland K.H., Rotter V. (2011). Amplification of the 20q chromosomal arm occurs early in tumorigenic transformation and may initiate cancer. PLoS One.

[77] Lorenzetto E., Brenca M., Boeri M., Verri C., Piccinin E., Gasparini P., Facchinetti F., Rossi S., Salvatore G., Massimino M. (2014). YAP1 acts as oncogenic target of 11q22 amplification in multiple cancer subtypes. Oncotarget.

[78] Kehrmann A., Truong H., Repenning A., Boger R., Klein-Hitpass L., Pascheberg U., Beckmann A., Opalka B., Kleine-Lowinski K. (2013). Complementation of non-tumorigenicity of HPV18-positive cervical carcinoma cells involves differential mRNA expression of cellular genes including potential tumor suppressor genes on chromosome 11q13. Cancer Genet..

[79] Mazumder Indra D., Mitra S., Roy A., Mondal R.K., Basu P.S., Roychoudhury S., Chakravarty R., Panda C.K. (2011). Alterations of ATM and CADM1 in chromosomal 11q22.3–23.2 region are associated with the development of invasive cervical carcinoma. Hum. Genet..

[80] Huang K.F., Lee W.Y., Huang S.C., Lin Y.S., Kang C.Y., Liou C.P., Tzeng C.C. (2007). Chromosomal gain of 3q and loss of 11q often associated with nodal metastasis in early stage cervical squamous cell carcinoma. J. Formos. Med. Assoc..

[81] Rizvi M.M., Alam M.S., Mehdi S.J., Ali A., Batra S. (2012). Allelic loss of 10q23.3, the PTEN gene locus in cervical carcinoma from Northern Indian population. Pathol. Oncol. Res..

[82] Wang S., Li Y., Han F., Hu J., Yue L., Yu Y., Zhang Y., He J., Zheng H., Shi S., Fu X., Wu H. (2009). Identification and characterization of MARVELD1, a novel nuclear protein that is down-regulated in multiple cancers and silenced by DNA methylation. Cancer Lett..

[83] Poignée M., Backsch C., Beer K., Jansen L., Wagenbach N., Stanbridge E.J., Kirchmayr R., Schneider A., Dürst M. (2001). Evidence for a putative senescence gene locus within the chromosomal region 10p14-p15. Cancer Res..

[84] Amiel A., Kolodizner T., Fishman A., Gaber E., Klein Z., Beyth Y., Fejgin M.D. (1998). Replication pattern of the p53 and 21q22 loci in the premalignant and malignant stages of carcinoma of the cervix. Cancer.

[85] Simpson S., Woodworth C.D., DiPaolo J.A. (1997). Altered expression of Erg and Ets-2 transcription factors is associated with genetic changes at 21q22.2–22.3 in immortal and cervical carcinoma cell lines. Oncogene.

[86] Lennerz J.K., Perry A., Mills J.C., Huettner P.C., Pfeifer J.D. (2009). Mucoepidermoid carcinoma of the cervix: another tumor with the t(11;19)-associated CRTC1-MAML2 gene fusion. Am. J. Surg. Pathol..

[87] Miyai K., Furugen Y., Matsumoto T., Iwabuchi K., Hirose S., Kinoshita K., Fujii H. (2004). Loss of heterozygosity analysis in uterine cervical adenocarcinoma. Gynecol. Oncol..

[88] Engelmark M.T., Ivansson E.L., Magnusson J.J., Gustavsson I.M., Wyöni P.I., Ingman M., Magnusson P.K., Gyllensten U.B. (2008). Polymorphisms in 9q32 and TSCOT are linked to cervical cancer in affected sib-pairs with high mean age at diagnosis. Hum. Genet..

[89] Jee K.J., Kim Y.T., Kim K.R., Aalto Y., Knuutila S. (2001). Amplification at 9p in cervical carcinoma by comparative genomic hybridization. Anal. Cell Pathol..

